# Dispersal and diversity in the earliest North American sauropodomorph dinosaurs, with a description of a new taxon

**DOI:** 10.1098/rspb.2010.1867

**Published:** 2010-10-06

**Authors:** Timothy B. Rowe, Hans-Dieter Sues, Robert R. Reisz

**Affiliations:** 1 Jackson School of Geosciences, C1100, The University of Texas at Austin, Austin, TX 78712, USA; 2 Vertebrate Paleontology Laboratory, The University of Texas at Austin, Austin, TX 78712, USA; 3 Department of Paleobiology, National Museum of Natural History, Smithsonian Institution, MRC 121, PO Box 37012, Washington, DC 20013-7012, USA; 4 Department of Biology, University of Toronto Mississauga, Mississauga, ON, CanadaL5L 1C6

**Keywords:** Dinosauria, Sauropodomorpha, extinction, vicariance, dispersal, phylogeny

## Abstract

Sauropodomorph dinosaurs originated in the Southern Hemisphere in the Middle or Late Triassic and are commonly portrayed as spreading rapidly to all corners of Pangaea as part of a uniform Late Triassic to Early Jurassic cosmopolitan dinosaur fauna. Under this model, dispersal allegedly inhibited dinosaurian diversification, while vicariance and local extinction enhanced it. However, apomorphy-based analyses of the known fossil record indicate that sauropodomorphs were absent in North America until the Early Jurassic, reframing the temporal context of their arrival. We describe a new taxon from the Kayenta Formation of Arizona that comprises the third diagnosable sauropodomorph from the Early Jurassic of North America. We analysed its relationships to test whether sauropodomorphs reached North America in a single sweepstakes event or in separate dispersals. Our finding of separate arrivals by all three taxa suggests dispersal as a chief factor in dinosaurian diversification during at least the early Mesozoic. It questions whether a ‘cosmopolitan’ dinosaur fauna ever existed, and corroborates that vicariance, extinction and dispersal did not operate uniformly in time or under uniform conditions during the Mesozoic. Their relative importance is best measured in narrow time slices and circumscribed geographical regions.

## 1. Introduction

Dinosauria originated in the Southern Hemisphere in the Middle or early Late Triassic, where it quickly diversified into its three major constituent clades, Ornithischia, Sauropodomorpha and Theropoda [1–3]. Matters of long-standing interest and current debate involve the phylogenetic relationships within these clades, and the timing and driving causes behind their distribution and diversity. Contemporary models often portray two broad episodes in Mesozoic dinosaur evolution [4–7]. The first played out in the Late Triassic and Early Jurassic, when the three major clades rapidly spread across Pangaea to establish a cosmopolitan community of uniform, low diversity. Ease of terrestrial dispersal across Pangaea is believed to have limited faunal differentiation that might otherwise have arisen in response to geographical isolation, explaining the range and apparent uniformity of this community. The second episode ensued as Pangaea fragmented and drifted apart during the Middle to Late Jurassic and the Cretaceous. Vicariance accelerated diversification through increased faunal isolation and provincialism, by regional extinction, and with episodic intercontinental ‘sweepstakes’ arrivals. In sum, three processes allegedly governed the pattern of dinosaur diversification. Vicariance and regional extinction generally enhanced diversity, while dispersal reduced it [4].

Considerable effort has been devoted to rigorous testing of these generalizations. Cladistic biogeographic methods testing congruence of clade splitting events with vicariant events provided a first approximation of the large-scale relationship of Pangaean fragmentation with dinosaurian diversification [4,5]. Successively refined phylogenetic analyses provided constraints on scenario-building as well as sources of hypotheses amenable to further testing [1–3]. Tree reconciliation analyses added statistical precision to measuring congruence of phylogenetic splitting events with continental fragmentation [8,9]. These studies found that vicariance is detectable statistically in Late Jurassic and Cretaceous dinosaur cladogenesis [9], in Cretaceous crocodyliforms, and in other late Mesozoic clades [10].

Unfortunately, the studies also showed that extinction and most forms of dispersal are resistant to statistical tests, and that phylogenetic analysis remains the best tool to assess their relative impact on dinosaur diversification prior to and in the earliest stages of Pangaean fragmentation. An important advance was the realization that scales of temporal and geographical range are critical in framing such analyses [8,11]. Because diversity and distribution patterns change in potentially independent modes, pattern comparison may be increasingly error-prone across broader temporal and geographical ranges. In other words, the veracity of broad global generalizations may be tested by measuring them in narrow time slices and restricted areas.

We explore these general questions of diversification using a ‘reductionist’ approach to the problem. We describe a new taxon from the Lower Jurassic Kayenta Formation of Arizona that is among the oldest North American sauropodomorphs, and analyse its relationships in a restricted basal segment of the sauropodomorph clade, over a limited ‘slice’ of geological time and in a bounded geographical region. This approach affords a nuanced view of early sauropodomorph evolution in North America, and a refined perspective on the relative importance of dispersal, extinction and vicariance on dinosaurian evolution throughout the Mesozoic.

## 2. Material and methods

Early dinosaurs are the subject of intense scrutiny by researchers applying phylogenetic analysis to individual specimens, in an attempt at rigorous enforcement of apomorphy-based taxonomic allocation of each catalogued specimen, to examine ‘microscopically’ each entry on the local faunal lists upon which more sweeping global statements stand [12–15]. The results produce opposing revisions to older measures of faunal composition, each with important corollaries.

Diversity in any given faunal assemblage is reduced when older identifications based on fragmentary specimens lacking diagnostic apomorphies are invalidated. For example, all three major dinosaurian clades are reported in Late Triassic North American tetrapod assemblages, but when the evidentiary standard of apomorphy is scrupulously enforced, theropods are the only dinosaurs verifiably recorded in North America at that time [12–15]. This begs the question of ‘primitive absence’ versus ‘pseudoabsence’ (i.e. present but undiscovered [9]). Nonetheless, these Triassic deposits are broadly exposed and have been intensely sampled by more than a century of fieldwork. Triassic sauropodomorphs verifiably occur in South America, Africa, Europe and East Greenland [16]. But at present, there is not a single known specimen preserving an apomorphy to refute the statement that ornithischians and sauropodomorphs were primitively absent in North America in the Triassic.

The opposite effect increases taxonomic resolution and diversity, where a taxon once believed wide-spread is shown to comprise separate diagnosable individuals. The Kayenta sauropodomorph was first referred to as ‘*Massospondylus* sp.’ in accord with the view that *Massospondylus* was part of a cosmopolitan Early Jurassic continental fauna [17–19]. However, thanks in part to new complete specimens, its distinctness is now evident. This increased resolution raises measures of sauropodomorph diversity in the Early Jurassic, while also decreasing the geographical ranges of individual taxa and confining *Massospondylus* to southern Africa. Both classes of revision cast new light on the biogeography and diversification of early dinosaurs.

Accepting the primitive absence of North American sauropodomorphs in the Triassic places their arrival on the continent in an entirely new temporal context of the Early Jurassic. In total, North American Early Jurassic sauropodomorphs are known from approximately 20 individual specimens that support the diagnosis of three nominal taxa. These are *Anchisaurus polyzelus* from the Portland Formation in the Hartford Basin [20,21], *Seitaad ruessi* from the Navajo Sandstone of Utah [22] and the Kayenta taxon named below. Additional material from the McCoy Brook Formation of Nova Scotia (Canada) probably represents a fourth taxon, but it remains undiagnosed [23]. The McCoy Brook specimens represent the oldest known record (Hettangian) of sauropodomorphs in North America. *Anchisaurus* and the Kayenta taxon are younger (Sinemurian–Pliensbachian), and *Seitaad* is the youngest (Toarcian).

**Figure 1.**
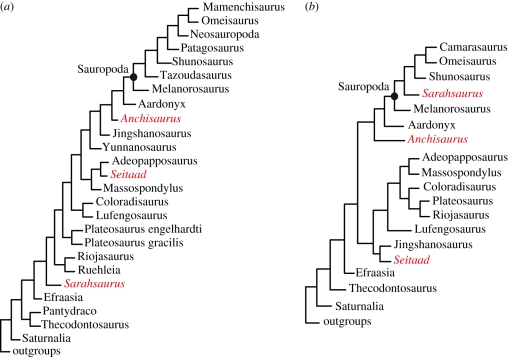
Phylogenetic relationships of North American sauropodomorphs (highlighted in red), based on pruned matrices omitting all taxa less then 50% complete (except *Seitaad*) from (*a*) the augmented Yates [24] matrix and (*b*) the Upchurch *et al*. [25] matrix. Total evidence results including strict, 50% majority, and Adams consensus trees are detailed in the electronic supplementary material.

Did North American sauropodomorphs arrive in a single sweepstakes event to undergo a local adaptive radiation, or did they arrive in multiple dispersal events? To answer this question, we added the Kayenta taxon to two different published taxon/character matrices focused on basal sauropodomorph relationships [24,25]. The augmented ‘Yates matrix’ [24] consists of 51 taxa and 361 characters, and includes the more recently named Early Jurassic taxa *Glacialisaurus* (Antarctica; [26]), *Adeopapposaurus* (Argentina; [27]) and *Seitaad* [22], plus several new characters. The augmented ‘Upchurch *et al*. matrix’ [25] consists of 38 taxa, including *Adeopapposaurus* and *Seitaad*, and 292 characters. We analysed both matrices using PAUP* 4.0a11 Beta [28] and evaluated character distributions with MacClade [29]. Analyses were run using a heuristic search with maxtree set at 10 000, TBR branch swapping and DELTRAN optimization settings. Multi-state characters were unordered and polymorphism treated as uncertainty (figure 1).

Our analysis had two primary goals. The first was to verify that the Kayenta taxon is diagnosable, and the second was to examine narrowly its relatedness to other North American Early Jurassic sauropodomorphs. A sweepstakes arrival into North America would link them in a clade as closest relatives, whereas separation on the tree would indicate multiple dispersals.

We ran three sets of analyses with each matrix. The first two were ‘total evidence’ parsimony analyses for all taxa and characters. The first was unrooted and the second was rooted using outgroups specified by Yates [24] and Upchurch *et al*. [25]. The Yates matrix was insensitive to rooting, and both analyses yielded 130 equally parsimonious trees of 1234 steps. The Upchurch *et al.* matrix was sensitive to rooting, and both analyses yielded 3585 trees of 820 steps. With PAUP, we generated strict, 50 per cent majority, and Adams consensus trees (electronic supplementary material, figures S1–S6). Using REDCON 3.0 [30], we generated reduced cladistic consensus trees (electronic supplementary material, tables S1 and S2).

Owing to discordant tree topologies between the two matrices, we conducted a third set of analyses that involved taxon pruning, to test whether taxon sampling and missing data affected our results. We first tested ‘safe taxonomic reduction’ [31] using TAXEQ3 [32], which indicated that no ‘equivalent taxa’ susceptible to ‘safe’ (*a priori*) removal were present in either matrix. We then ran a series of *a posteriori* pruning experiments based on percentages of missing data [33,34]. The taxa ranged from 99 to only 4 per cent complete (electronic supplementary material, tables S3 and S4). Starting with each ‘total matrix’, we sequentially eliminated incomplete taxa in both the ingroup and outgroup, until all taxa less than 50 per cent (an arbitrary limit) were excluded. The only exception was *Seitaad*, which fell below this threshold. As a North American taxon it was retained in all analyses (figure 1).

### (a) CT imaging

To augment conventional preparation, we scanned the holotype braincase at The University of Texas High Resolution X-ray Computed Tomography Facility (http://www.digimorph.org/specimens/Sarahsaurus_aurifontanalis/).

## 3. Systematic palaeontology

 Dinosauria Owen 1842

  Saurischia Seeley 1887

   Sauropodomorpha Huene 1932

    *Sarahsaurus aurifontanalis* taxon nov.

### (a) Etymology

Named in honour of Sarah (Mrs Ernest) Butler, whose broad interests in the arts, the sciences and medicine have enriched Texas in so many marvellous ways, and *sauros* (Gr., lizard); second part of binomen from *aurum* (L., gold) and *fontanalis* (L., of the spring), in reference to Gold Spring, Arizona, where the holotype was discovered.

### (b) Holotype

**Figure 2.**
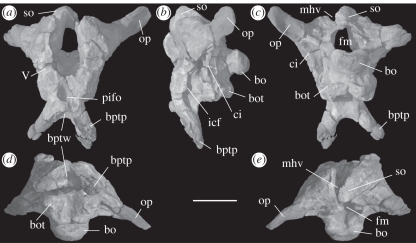
*Sarahsaurus aurifontanalis*, holotype (TMM43646-2). Three-dimensional reconstructions from HRXCT of the braincase (see http://www.digimorph.org/specimens/Sarahsaurus_aurifontanalis/) in (*a*) anterior, (*b*) left lateral, (*c*) posterior, (*d*) ventral and (*e*) dorsal views. bo, basioccipital; bot, basicranial tuber; bptp, basipterygoid process; bptw, wall between basipterygoid processes; ci, crista interfenestralis; fm, foramen magnum; icf, foramen for internal carotid artery; mhv, canal for middle cerebral vein; op, opisthotic-exoccipital; pifo, pituitary fossa; so, supraoccipital; V, trigeminal nerve foramen. Scale bar, 2 cm.

**Figure 3.**
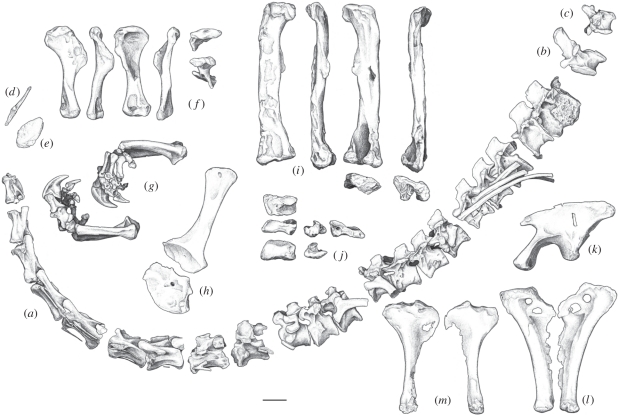
*Sarahsaurus aurifontanalis*, holotype (TMM 43646-2): (*a*) presacral vertebral column from vertebrae 2 to 22; (*b*) second caudal vertebra; (*c*) mid-caudal vertebra; (*d*) left clavicle in medial view; (*e*) left sternal plate in ventral view; (*f*) right humerus in dorsal, lateral, ventral, medial, proximal and distal views; (*g*) right antebrachium and manus in lateral and medial views; (*h*) left scapula and coracoid; (*i*) right femur in anterior, medial, posterior, lateral, proximal and dorsal views; (*j*) right astragalus in distal, posterior, ventral, medial, lateral and anterior views; (*k*) left ilium in lateral view; (*l*) pubes in anterior view and (*m*) left and right ischia, in lateral view. Scale bar, 5 cm.

TMM 43646-2, a partial skull (premaxilla, frontal, quadrate and braincase) and a nearly complete, largely articulated postcranial skeleton (figures 2 and 3). The holotype is ontogenetically mature based on fusion of the opisthotic-exoccipitals to the basioccipital, fusion of the neural arches to their centra along the entire vertebral column, and fusion of the sacral and caudal ribs to their respective vertebrae.

### (c) Locality and horizon

Northern edge of Gold Spring Wash drainage basin, in northeastern Arizona, USA; middle third of the ‘Silty Facies’ of the Kayenta Formation (Glen Canyon Group); Early Jurassic (Sinemurian–Pliensbachian).

### (d) Referred specimens

**Figure 4.**
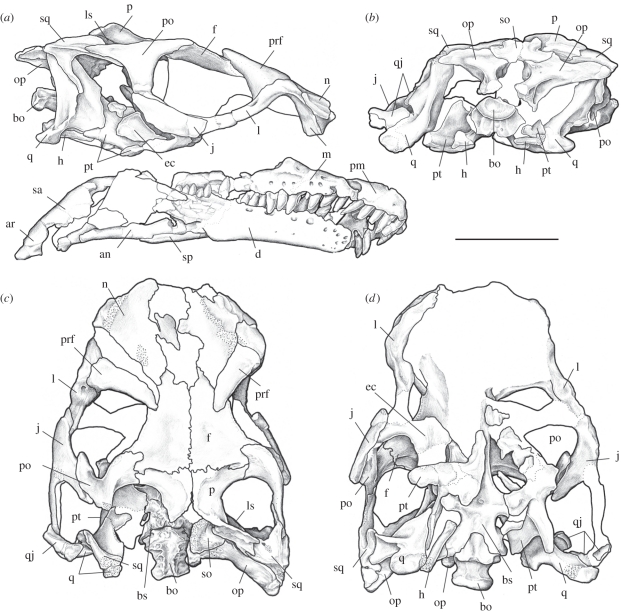
*Sarahsaurus aurifontanalis*, referred skull (MCZ 8893), in (*a*) lateral, (*b*) occipital, (*c*) dorsal and (*d*) ventral views. an, angular; ar, articular; bo, basioccipital; bs, basisphenoid; ec, ectopterygoid; f, frontal; h, hyoid; j, jugal; l, lacrimal; ls, laterosphenoid; m, maxilla; n, nasal; op, opisthotic-exoccipital; p, parietal; po, postorbital; prf, prefrontal; pm, premaxilla; pt, pterygoid; q, quadrate; qj, quadratojugal; so, supraoccipital; sq, squamosal. Scale bar, 5 cm.

TMM 43646-3, a partial postcranial skeleton from the holotype quarry; and Harvard University, Museum of Comparative Zoology, MCZ 8893 (figure 4), a crushed but nearly complete skull and mandible, with cervical and caudal vertebral fragments, the distal end of a humerus and a femoral shaft. Referral of the MCZ specimen is based on skeletal elements shared with the holotype, including the braincase, quadrate, frontal, premaxilla, cervical vertebrae and humerus, in which the two specimens are identical in all character scores and in all other respects. The only notable difference is that MCZ 8893 represents a less mature individual, with open sutures between the exoccipital-opisthotics and basioccipital, whereas the holotype was fully mature and has closure of these sutures.

### (e) Diagnosis

Among sauropodomorphs, *Sarahsaurus* is unique in the configuration of a low wall between the basicranial tubera with a central anterior fossa; presence of spine tables on its dorsal vertebrae; a manual phalangeal formula of 2-3-4-2-2; and presence of a pubic foramen. *Sarahsaurus* is also diagnosed by the largest unique suite of character states of any taxon analysed in either matrix. Our diagnoses are based on the pruned analysis, because unequivocal local apomorphic states cannot be taken from consensus trees [31]. In the pruned Yates matrix, *Sarahsaurus* had a branch length (BL) of 61 steps (electronic supplementary material, table S5) and in the Upchurch *et al*. matrix 35 steps (electronic supplementary material, table S6). Most characters are homoplastic with taxa elsewhere on the tree, but the combinations were unique in all tests.

### (f) Description

Here, we enumerate derived character states that bear on the Placement of *Sarahsaurus* among sauropodomorphs. The external narial margin is formed by the premaxilla and nasal, with a small contribution from the maxilla. Behind the premaxillary teeth, a posterolateral process overlaps the maxilla along the posteroventral corner of the naris. The palatal shelves of the premaxillae are narrow and enclose an incisive foramen between them. A large subnarial foramen penetrates the descending segment of the suture between the premaxilla and maxilla above the tooth row, and a small neurovascular foramen is situated above the alveolar margin behind the first tooth on the right premaxilla.

The maxilla has a subnarial ramus that is longer than deep. A shelf-like area lateral to the external naris extends onto the anterior end of the maxilla. There are at least seven distinct neurovascular foramina on the lateral surface of the maxilla, the last of which opens posteriorly. The medial shelves of the maxillae were probably in contact anteriorly.

The nasal has a posterior process extending between the frontal and prefrontal. The nasal contributes to the lateral edge of the antorbital fossa dorsally but does not form a deep recess over the dorsal apex of the fossa. The length of the antorbital fenestra is less than that of the orbit. The jugal makes a small contribution to the border of the antorbital fenestra. The length of the dorsal process of the lacrimal is less than half the height of its ventral process. The antorbital fossa extends onto the ventral end of the lacrimal. The prefrontal has a long ventral process that extends medial to the posterior foramen for the canal for the nasolacrimal duct, and it medially overlaps the ventral ramus of the lacrimal. The prefrontal is more extensively exposed on the skull roof than the lacrimal. There is no ridge on the dorsolateral surface of the lacrimal and no associated knob on the lateral aspect of the prefrontal.

The frontal is longer than wide and broadly contributes to the orbital rim. It is gently concave in the interorbital region, which is constricted at mid-length. The frontal does not enter into the anterior margin of the supratemporal fenestra, but the supratemporal fossa extends for a short distance onto its posterodorsal portion. The anterior margin of the infratemporal fenestra continues below the orbit. The anterior process of the postorbital is forked at its medial contact with the frontal, and the supratemporal fossa extends onto its posterodorsal surface. The ventral process of the postorbital overlaps the dorsal process of the jugal, whereas its posterior process overlaps the anterior process of the squamosal.

The posterolateral process of the parietal is deflected ventrolaterally and contacts the medial process of the squamosal slightly below the top of the skull roof. The ventral process of the squamosal is strap-like and four times as long as its basal width. The quadrate foramen penetrates the suture between the quadrate and the quadratojugal. The angle between the anterior and the dorsal rami of the quadratojugal is acute (approx. 60°).

The braincases of the holotype and the referred skull correspond in detail and share the unique configuration of a shallow transverse wall between the basipterygoid processes. There is a large postparietal fenestra between the parietal and the supraoccipital. The supraoccipital is diamond-shaped and inclined at 45° so that its anterior tip lies above the basipterygoid process. The distinct basipterygoid processes are connected only by a narrow transverse ridge. The floor of the braincase is relatively straight with the basal tubera, basipterygoid processes and parabasisphenoid rostrum all more or less aligned. A ridge forms along the junction of the parabasisphenoid and the basioccipital, between the basal tubera, and has a smooth anterior face. Co-ossification at the extremities of the basal tubera is complete in the holotype, so that the basioccipital and the parabasisphenoid form a single rugose tuber on either side. The basal tubera are knob-like, with the basisphenoid component protruding rostral to the lateral basioccipital components.

The jugal process of the ectopterygoid is strongly recurved and hook-like. The medial process of the pterygoid is flat and blunt. The dentary curves ventrally towards its anterior tip, and there is no evidence of a rhamphotheca. The jaw articulation lies at a level below the dorsal margin of the dentary. The referred skull was broken across the palate and few other details are visible.

The dentition is moderately heterodont. The maxillary tooth row extends posteriorly beyond the posterior end of the dentary tooth row. There are four premaxillary teeth, 16 maxillary teeth and 20 dentary teeth. The first dentary tooth is inset a short distance from the anterior tip of the dentary and is slightly procumbent. Individual tooth crowns are labiolingually compressed, taller apicobasally than wide and convex to varying degrees mesiodistally, straight rather than recurved and more or less symmetrical in labial view. The tooth crown and root are separated by a slight constriction. The mesial and the distal carinae are coarsely serrated with denticles that project apically at an angle of about 45° relative to the carina, as in other basal sauropodomorphs. There are up to 20 denticles per tooth crown. The crowns are angled relative to the long axis of the jaw and imbricate slightly, such that each tooth has its mesial margin lying lingual to the distal margin of the crown immediately in front.

*Sarahsaurus* has 10 cervical vertebrae, 14 dorsals, three sacrals and approximately 50 caudals. The cervical postzygapophyses are flush with the ends of their centra, while the prezygapophyses extend forward over the preceding centra to reach their articulations. The cervical vertebrae are laterally compressed. On all presacral vertebrae, there is a centrodiapophyseal lamina. The epipophyses do not overhang the posterior margin of the postzygapophyses.

The lateral surfaces of the dorsal centra are not deeply excavated for presumed air sacs. Laterally expanded tables are present at mid-length on the distal apices of the cervical and the anterior dorsal neural spines. The posteriormost presacral rib is fused to its vertebra. In the sacrum, it is unclear whether the third sacral vertebra represents a dorsosacral or a caudosacral. The distal ends of the sacral ribs are fused into a sacricostal yoke that attaches to the ilium. A longitudinal sulcus is present on the ventral surfaces of the caudal centra.

The scapular blade is hourglass-shaped, with narrow, curved margins in its mid-section. The coracoid has a flat surface between the glenoid and the coracoid tubercle on its posteroventral edge. The scapula and coracoid are separated (figure 3*h*), and the edges along which they abut one another are slightly incised, suggesting the persistence of cartilage in this zone. The more basal sauropodomorph *Saturnalia* preserves the ancestral condition for Saurischia in that the scapula and coracoid are fused in mature individuals. In the holotype of *S. aurifontanalis*, the lack of suturing between the scapula and the coracoid may indicate that skeletal maturation, though largely finished, was not complete at the time of death.

One of the paired sternal plates was recovered, and it bears a longitudinal ridge with an ovoid articular surface for the clavicle (figure 3*e*). The clavicle is a long, straight bone with a broad articular facet for the sternal plate, in what was evidently a synovial joint (figure 3*d*). An elongate process extends from this articulation towards the midline, where it met its counterpart. The right and left clavicles did not fuse, but were apparently held in close proximity by a median ligament.

The humerus (figure 3*f*) is more than half as long as the femur. The deltopectoral crest is long, extending half the length of the humerus, and its leading edge is sigmoidal. The proximal end of the ulna is triradiate and incised by a notch for reception of the head of the radius (figure 3*g*). In the wrist, the maximum linear dimensions of the ulnare and radiale are less than those of any of the distal carpals. The first distal carpal is wider than the transverse width of metacarpal I. The lateral end of the first distal carpal overlaps the second distal carpal. The second distal carpal does not completely cover the proximal end of the second metacarpal. In the manus, there is strong asymmetry in the lateral and medial condyles of the first metacarpal. The ungual phalanx on digit I is the largest in the hand. Metacarpal V has a broad proximal end that is nearly as wide as long with a strongly convex proximal surface.

The iliac portion of the acetabulum is completely open (figure 3*k*). The ischial peduncle of the ilium is reduced and about half the length of the pubic peduncle, and has a posteriorly projecting heel at its distal end. The pubic shaft supports a broad, thin apron of bone that has a concave profile. The pubis (figure 3*l*) is unique among sauropodomorphs in the presence of both an obturator foramen and a pubic foramen, whereas in most archosaurs only the obturator foramen is present. The pubis makes more than twice the contribution to the acetabulum than the ischium. At its distal end, the ischium expands dorsoventrally to nearly twice the thickness of the shaft at its isthmus (figure 3*m*).

The femoral shaft (figure 3*i*) is straight and has an elliptical transverse section. The long axis of the femoral head and the transverse axis of the distal end are roughly parallel. Both the fourth trochanter and the anterior trochanter are low ridges. On the distal end of the femur is a depression for the leg extensor musculature. The fibula has expanded proximal and distal ends, and bears a bulbous muscle scar on its lateral surface. The length of the tibia is about 84 per cent the length of the femur. The transverse width of the distal end of the tibia is subequal to its anteroposterior width. Its medial malleolus is reduced, exposing the posterior fossa of the astragalus in posterior view. The ascending process of the astragalus (figure 3*j*) keys into the distal end of the tibia.

In the pes, both the medial and lateral margins of the proximal end of metatarsal II are concave. Metatarsal V has a transversely broad proximal end and narrow distal end so that the metatarsal is funnel- or paddle-shaped.

## 4. Discussion

### (a) Phylogenetic results

The two matrices produced different trees under total evidence analyses. The Yates matrix yielded consistent relationships among the North American taxa under all conditions (electronic supplementary material, figures S1–S3). *Anchisaurus* was positioned closest to Sauropoda, whereas *Seitaad* clustered with *Adeopapposaurus* and *Massospondylus* in a more basal clade. *Sarahsaurus* was the most basal of the three, lying on its own branch in the Adams consensus tree, or clustered with *Glacialisaurus*, *Lufengosaurus* and *Coloradisaurus* in the 50 per cent majority consensus tree.

The Upchurch *et al*. matrix also produced consistent relationships among the North American taxa in the total evidence analyses (electronic supplementary material, figures S4–S6). *Anchisaurus*/*Ammosaurus* (coded separately) formed a clade positioned closest to Sauropoda, whereas *Sarahsaurus* and *Seitaad* were successively more basal. Both the Adams and 50 per cent majority consensus trees clustered *Sarahsaurus* in an unresolved clade with *Adeopapposauru*, *Massospondylus, Lufengosaurus, Coloradisaurus, Mussaurus* and *Plateosaurus. Seitaad* was linked with *Jingshanosaurus* in a more basal clade in the 50 per cent majority consensus tree.

Both matrices were sensitive to taxon pruning. The Yates matrix was the most robust, and removing all taxa less than 50 per cent complete slightly altered relationships among Sauropoda without changing the relative positions of the North American taxa (figure 1*a*). In the pruned Upchurch *et al*. matrix, *Sarahsaurus* moved to a basal position within Sauropoda, whereas *Anchisaurus* and *Seitaad* remained outside and were successively more basal (figure 1*b*).

Which of these results should we accept? In response to our first question, all tests confirm *Sarahsaurus* as diagnosable based on autapomorphies plus a unique suite of character-states. The exact combination of states varied between trees. Because unambiguous apomorphy lists cannot be generated from consensus trees, we used results of the pruned analysis as the basis for separate ‘Yates’ and ‘Upchurch *et al*.’ diagnoses (electronic supplementary material, tables S5 and S6).

In response to the second question, all tests rejected the hypothesis of a local adaptive radiation and were consistent with the interpretation that sauropodomorphs populated North America in at least three independent dispersal events during the Early Jurassic. Thus, all results provide decisive answers to our first two questions.

The results were less satisfying in resolving the important (if, in this context, peripheral) problem of basal sauropodomorph relationships generally. The Yates matrix produced a highly resolved, relatively stable tree in all three analyses. However, it presents one potentially impeachable result in placing *Sarahsaurus* as the most basal North American sauropodomorph. There, *Sarahsaurus* sits isolated on a branch 61 steps in length, with 44 character states in the vertebral column, girdles and limbs shared homoplastically with Sauropoda, plus 14 reversals from derived (1 or 2) to ancestral (0) states (electronic supplementary material, table S5). Taxon-pruning experiments have shown a long BL comprised mostly of homoplasies and reversals to be symptomatic of topological error [33,34]. This, together with its distribution in time (approx. 40 Myr younger than the oldest known sauropodomorph) and in space (remotely situated in the Western Interior), suggests that the basal placement of *Sarahsaurus* is probably incorrect.

In the pruned Upchurch *et al*. matrix, placement of *Sarahsaurus* within Sauropoda cut its BL almost in half, and the reversals and homoplasies are distributed more widely across the tree. It may be prejudicial that this unique result obtained by pruning taxa, because even incomplete taxa can provide phylogenetic signal [31,33,34], and five of our six analyses reached different conclusions. Nevertheless, the many striking resemblances that *Sarahsaurus* shares with giant sauropods lend a measure of credibility to this result. It is also consistent with recent studies suggesting that skeletal features once believed tied to gigantism, such as columnar hindlimbs, instead originated in smaller animals for other functions and only later facilitated the evolution of gigantic size [20,35]. A robust solution to basal sauropodomorph phylogeny awaits the recovery of more complete fossils.

### (b) Vicariance, extinction and dispersal

Vicariance predicts patterns of ‘foreign relationships’ resembling that of the Early Jurassic North American sauropodomorphs, but Pangaean fragmentation was only incipient at this time and could not have produced this pattern [36]. However, the multiple independent arrivals of sauropodomorphs in North America are consistent with an ‘area coalescence’ model in which taxa from separate geographical areas come together by dispersing into a newly accessible region [8–10]. Like vicariance, this allows taxonomically diverse groups to effect similar changes in range. The coalescence event in this case was not a colliding tectonic plate or the elimination of an oceanic barrier, but more probably involved easement of physical barriers to dispersal from the adjoining lands of present-day South America, Europe and Africa, where the phylogenetic affinities of the North American sauropodomorphs probably lie. Also implicated by some, but not all, of the phylogenetic results is the establishment of a land connection to eastern Asia.

Corroborating the area coalescence model is repetition of the pattern of foreign relationships by ornithischian dinosaurs. Also apparently absent in the North American Triassic [12–14], their oldest representatives appear simultaneously with *Sarahsaurus* in the Kayenta fauna and include the thyreophorans *Scutellosaurus lawleri* [37] and ‘*Scelidosaurus* sp.’ [38] and an undescribed heterodontosaurid [39]. A recent phylogenetic hypothesis of ornithischian interrelationships [40] would suggest that all three Kayenta taxa are related more closely to foreign ornithischians than to each other. Kayenta tritylodontid cynodonts [41] and a goniopholidid crocodyliform [42] also repeat this pattern of apparent immigration.

The Central Atlantic Magmatic Province (CAMP) is implicated in shaping these events, based on its timing and geographical position. This large igneous province is represented by tholeiitic lava flows, dikes and sills in eastern North America, northern South America, northwestern Africa and western Europe, in a broad band separating the North American interior from most of the rest of Pangaea [43]. High-precision geochronology indicates that CAMP activity occurred in a brief magmatic episode all along the pre-Atlantic rift zone approximately 201.5 Ma, and appears to be temporally coincident with end-Triassic extinctions in marine faunas [44,45]. CAMP volcanism may have surpassed even the end-Permian Siberian flood basalts in volume and area, profoundly altering climate, and disrupting Pangaea by opening the proto-Atlantic Ocean [43–46].

The timing of events suggests that in North America, at least, the end-Triassic extinctions were not driven by competitive invasion of foreign taxa, nor is there faunal evidence of such an invasion. Theropods were present during most or all of the Late Triassic and survived the extinction [15]. But it may not have been until after the end-Triassic extinctions, cessation of CAMP volcanism and following an early Hettangian ‘recovery period’ of up to 2 Ma [44], that sauropodomorphs and other members of its Early Jurassic fauna independently dispersed into North America. Current reconstructions of Early Jurassic Pangaea [47] suggest the possibility of terrestrial dispersal from South America, Africa, Europe and possibly Asia into North America. This ‘local snapshot’ is consistent with a broader picture of dinosaurs as opportunistic occupants of niches vacated by prior extinctions [3,5,14,15,48,49].

However, our snapshot contradicts the assertion that dispersal reduced diversity [3], or was less influential than vicariance in shaping dinosaurian diversity. All three early dinosaurian clades reflect high degrees of endemism [12–15] that contest the notion of a uniform cosmopolitan dinosaur community in the Late Triassic and Early Jurassic, or at any time up to the present day. The ‘cosmopolitan dinosaur community’ is more probably an artefact of poor taxonomic resolution, and confusing the evolutionary process of divergence with the historical result of accumulated morphological novelty. Late Jurassic and Cretaceous dinosaurian faunas are sharply differentiated by the discrete landmasses they occupy, and easily recognizable owing to more than 100 Ma of accumulated novelty and divergence. The Late Triassic and Early Jurassic patterns are more subtle and lack sharp geographical boundaries, but are present nevertheless.

The earliest North American sauropodomorphs support the view that early dinosaurian diversification was driven by dispersal and adaptation over the vast and ecologically heterogeneous environs of Pangaea, and opportunistically amplified by the end-Triassic extinctions. Only later in the Jurassic, as Pangaea disintegrated, was vicariance superimposed as a diversification factor. It remains to be seen whether this new factor or dispersal on continental scales had greater impact at any given time.

The relative importance of competition, vicariance, extinction and dispersal often is framed as an essentialist debate promoting a single dominant cause throughout dinosaurian history. It seems more likely that these factors did not operate uniformly over time or under uniform conditions, and only in narrow time slices and bounded regions can their roles be assessed.
